# 3D-architected acceptor affords efficient, stable and stretchable photovoltaics

**DOI:** 10.1093/nsr/nwaf054

**Published:** 2025-02-22

**Authors:** Jin-Woo Lee, Jung-Yong Lee, Bumjoon J Kim

**Affiliations:** Department of Chemical and Biomolecular Engineering, Korea Advanced Institute of Science and Technology (KAIST), South Korea; School of Electrical Engineering, Korea Advanced Institute of Science and Technology (KAIST), South Korea; Department of Chemical and Biomolecular Engineering, Korea Advanced Institute of Science and Technology (KAIST), South Korea

Polymer solar cells (PSCs) have emerged as a transformative photovoltaic technology, offering inherent advantages such as light weight, flexibility, cost-effectiveness and environmental friendliness [1]. These attributes make PSCs particularly suitable for applications in wearable devices. Over the past decades, the evolution from fullerene-based acceptors to diverse small-molecule acceptors (SMAs) has been driven by innovative molecular design and in-depth understanding of the structure-performance relationships [2]. This progress has propelled PSCs to achieve unprecedented power conversion efficiencies (PCEs) beyond 20%. Yet, challenges related to stability and mechanical durability continue to hinder their path toward commercial application [3].

Recently, oligomerization of SMAs has been shown to effectively elevate the glass transition temperature (*T*_g_) of these materials, delaying molecular diffusion kinetics and suppressing thermodynamically driven phase segregation [4,5]. Unfortunately, the inherent rigidity of oligomeric SMAs creates hard, isolated aggregated structures, resulting in the brittleness of blend films in PSCs [6]. The aggregated structures in blend films can be tailored by fine-tuning the regioregularity, molecular weight and the conjugated molecular structures [7]. Typically, this aggregated structure consists of hard lamellar crystalline domains interspersed with rubbery amorphous phases. Under mechanical deformation, the amorphous phases play a crucial role in dissipating mechanical stress, thereby promoting strain hardening and improving film toughness [8,9]. Therefore, the key to improving mechanical resilience of these blend films, without compromising PCE and long-term stability, lies in precisely regulating the aggregated structure of the acceptor component and the intermixed domains with the donor materials in blend films.

**Figure 1.**
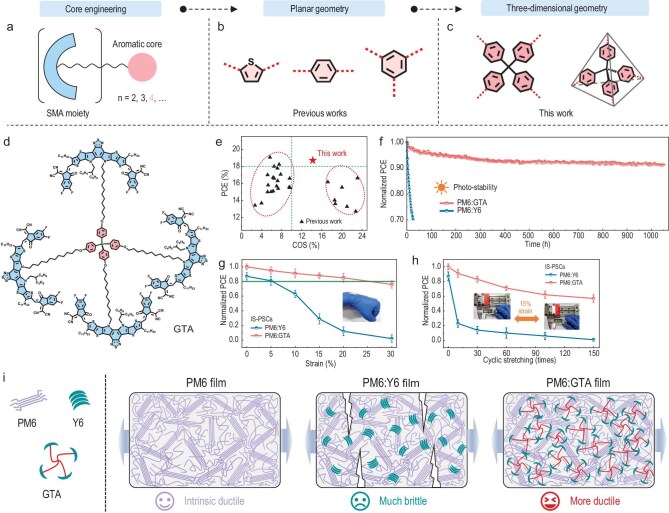
(a) Design rationales of tethered SMAs. The conceptual schemes of core engineering with (b) planar geometry and (c) three-dimensional geometry. (d) Molecular structure of GTA. (e) The statistical PCE and COS values of binary PSCs in this work and those of reported. (f) MPP stability test of the PM6:Y6 and PM6:GTA–based devices under 1-Sun equivalent illumination from white LEDs at the MPP conditions in open-air. (g) Plots of normalized PCE values of intrinsically stretchable PSCs under various strains. (h) Plots of normalized PCE values of intrinsically stretchable PSCs with stretching cycles under 15% strain. (i) Schematic illustration of the morphological features and crack states of PM6, PM6:Y6 and PM6:GTA films under external stress. Reproduced from Ref. [10] with permission.

In a recent study published in *National Science Review*, Prof. Zhiguo Zhang's group from Beijing University of Chemical Technology, in collaboration with Prof. Long Ye's group from Tianjin University, suggests a new tethered SMA design (Fig. 1) that simultaneously achieves the high PCE, morphological stability and mechanical robustness in PSCs [10]. Unlike traditional oligomeric acceptors linked via stiff end-groups, their newly designed tethered acceptor, GTA, features flexible alkyl chain linkages arranged in a tetrameric geometry. The key to this design is using tetraphenylmethane as the linking core to create (1) a three-dimensional structure that increases free volume and (2) a high C2 symmetry that preserves fast charge transport, thereby enhancing the mechanical robustness of blend films while retaining excellent electrical properties. As demonstrated by dynamic mechanical analysis and film-on-elastomer characterizations, this unique structural design preserves sub-*T*_g_ relaxation dynamics while forming a mechanically resilient yet dynamically adaptive network. As a result, intrinsically stretchable PSCs incorporating GTA exhibit outstanding mechanical robustness, retaining 88% of their initial PCE at 15% strain and 76% after 150 fatigue cycles. In addition, the GTA-based PSCs demonstrate excellent PCE and exceptional photostability. This study represents an excellent demonstration of a binary PSC system that simultaneously enhances efficiency, stability and stretchability through delicate molecular design. The tethered oligomerization approach provides a new pathway for addressing the long-standing challenges impacting the figure-of-merit in wearable devices.

In summary, this research highlights the potential of three-dimensional tethered oligomerization as a transformative strategy for intrinsically stretchable photovoltaics. With further development, this approach could accelerate the commercialization of wearable solar technologies and contribute significantly to the advancement of next-generation sustainable energy solutions.
